# Analysis of eye movements in the judgment of enjoyment and non-enjoyment smiles

**DOI:** 10.3389/fpsyg.2013.00659

**Published:** 2013-09-24

**Authors:** Melanie Perron, Annie Roy-Charland

**Affiliations:** Department of Psychology, Laurentian UniversitySudbury, ON, Canada

**Keywords:** enjoyment and non-enjoyment smiles, perceptual-attentional mechanisms, eye movements, facial expressions, smile judgment

## Abstract

Enjoyment smiles are more often associated with the simultaneous presence of the Cheek raiser and Lip corner puller action units, and these units' activation is more often symmetric. Research on the judgment of smiles indicated that individuals are sensitive to these types of indices, but it also suggested that their ability to perceive these specific indices might be limited. The goal of the current study was to examine perceptual-attentional processing of smiles by using eye movement recording in a smile judgment task. Participants were presented with three types of smiles: a symmetric Duchenne, a non-Duchenne, and an asymmetric smile. Results revealed that the Duchenne smiles were judged happier than those with characteristics of non-enjoyment. Asymmetric smiles were also judged happier than the non-Duchenne smiles. Participants were as effective in judging the latter smiles as not really happy as they were in judging the symmetric Duchenne smiles as happy. Furthermore, they did not spend more time looking at the eyes or mouth regardless of types of smiles. While participants made more saccades between each side of the face for the asymmetric smiles than the symmetric ones, they judged the asymmetric smiles more often as really happy than not really happy. Thus, processing of these indices do not seem limited to perceptual-attentional difficulties as reflected in viewing behavior.

The smile is often expressed during social interactions and represents a powerful signal that may serve an important purpose in affiliative behavior, cooperation, and social bonds (Tomkins, 1962; Owren and Bachorowski, 2001). While people smile when they are experiencing true enjoyment, due to their ability to control their facial movements and because of social requirements, smiles may also be expressed to conceal negative emotions, deceive other individuals, display positive affect in public situations or as a sign of politeness, shyness, or embarrassment (Tomkins, 1962; Ekman, 1993, 2001; Hess et al., 2002; Zaalberg et al., 2004; Ambadar et al., 2009). Consequently, in order to effectively respond to the presentation of smiles and better adapt to the situation, an individual would benefit from being able to identify and interpret signs of enjoyment smiles and those of other smiles. While this may seem ideal, data suggests that this ability is far from perfect.

Several recent studies have provided evidence that adults are sensitive to some characteristics that distinguish enjoyment and non-enjoyment smiles and that even adolescents and children as young as 5 and 6 years old are able to discriminate between types of smiles (Soppe, 1988; Frank et al., 1993; Gosselin et al., 2002a, 2010b; Thibault et al., 2009). However, such judgment remains challenging both for children and adults, performance being slightly above chance (Gosselin et al., 2002b; Chartrand and Gosselin, 2005; Del Giudice and Colle, 2007). In addition, little is known about the processes underlying the recognition of smiles and reasons explaining the difficulty encountered in their appreciation.

One hypothesis that has been put forward to explain the difficulty associated with smile recognition relies on perceptual and attentional mechanisms (Chartrand and Gosselin, 2005; Del Giudice and Colle, 2007; Boraston et al., 2008; Gosselin et al., 2010b; Manera et al., 2011). In effect, researchers have suggested that difficulties in distinguishing types of smiles might be attributable to problems seeing the relevant indices or the lack of attention to these cues that would help in their judgment. This hypothesis is a plausible explanation given that these indices have been reported as subtle, non-frequent and in some cases present in both situations: when the person is truly happy and when they are not (Ekman et al., 1981, 1988; Krumhuber and Manstead, 2009). However, recent data have shed doubt on the role of perceptual-attentional factors in the recognition of enjoyment smiles by showing no significant relationship between the smile judgment task and different measures of perceptual ability such as eye movements or action unit discrimination (Boraston et al., 2008; Manera et al., 2011). Nevertheless, other factors suggest that further research remains necessary before ruling out the importance of the perceptual-attentional mechanisms in the recognition of enjoyment and non-enjoyment smiles. For instance, data has not always been consistent across studies (Chartrand and Gosselin, 2005; Boraston et al., 2008; Gosselin et al., 2010b; Manera et al., 2011). In addition, research has mainly focused one of the cues that distinguish types of smiles (the Duchenne marker), while other morphological cues have received less attention. The goal of the current study was to investigate perceptual-attentional processes by examining eye movements during the judgment of smiles varying as a function of the Duchenne marker and symmetry.

## The duchenne marker

Research has shown that spontaneous smiles associated with felt enjoyment are characterized by the co-activation of the *Zygomatic Major* and the *Orbicularis Oculi* (Ekman et al., 1988, 1990; Duchenne, 1990; Ekman, 1992; Frank and Ekman, 1993). The Lip Corner Puller, or the Action Unit 12 (UA12) as described in the Facial Action Coding System (FACS, Ekman et al., 2002) stretches the lips outward and upward and creates the smile. The Cheek Raiser, known as the Duchenne marker or AU6 in the FACS, lifts the cheeks, pushes the skin surrounding the eye toward the eye socket narrowing the eye opening, bagging, or wrinkling the skin below the eye, and may cause crows' feet. Several studies have suggested that this type of smile is more likely to occur when an individual is exposed to pleasant stimulation as well as when positive subjective experience is self-reported (see e.g., Ekman et al., 1988, 1990; Frank et al., 1993; Soussignan and Schaal, 1996; Gonzaga et al., 2001).

More recently, new evidence has nuanced this close link between the Duchenne marker and the expression of enjoyment by showing that Duchenne smiles can also be expressed when participants are asked to pose smiles voluntary (Smith et al., 1996; Schmidt et al., 2006; Krumhuber and Manstead, 2009) or expressed even when people were under unpleasant circumstances (Keltner and Bonanno, 1997; Papa and Bonanno, 2008). Gosselin et al. (2010a) also found that 60% of adult participants were able to activate the Cheek Raiser voluntarily which contrasts with previous estimations reported in the literature (10–20%) (Ekman et al., 1980; Ekman and Friesen, 1982; Levenson et al., 1990).

Even if the Duchenne marker can arise from genuine happiness or from deliberate control, the literature provides evidence that this cue is used by perceivers to distinguish enjoyment from non-enjoyment smiles, especially in the western culture (Thibault et al., 2012). Adults use the Duchenne marker when they make judgments about several attributes concerning other people such as their personality, humor, and pleasantness (e.g., Frank et al., 1993; Soussignan and Schaal, 1996). Indeed, individuals displaying the contraction of the orbicularis oculi are generally considered more positively by others on those dimensions. Adults also use the Duchenne marker in smile judgment tasks; smiles containing this marker being consistently referred to with labels such as “true enjoyment,” “happier,” “real or felt,” “really happy,” or “more genuine” (Frank et al., 1993; Manera et al., 2011; Gosselin et al., 2002a,b; Chartrand and Gosselin, 2005; Leppänen and Hietanen, 2007; Miles and Johnston, 2007; Ambadar et al., 2009; Krumhuber and Manstead, 2009; Thibault et al., 2009, 2012; Calvo et al., 2013).

## The symmetry

The degree of symmetry of the expression is another morphological feature reported as distinguishing enjoyment and non-enjoyment smiles. Asymmetry is defined as the variation in expression intensity on one side of the face relative to the other side (Borod et al., 1997). While the literature suggests that the left side of the face is naturally more involved than the right side in the expression of emotions (Skinner and Mullen, 1991; Borod et al., 1997), with regards to smiles, research has shown that spontaneous smiles in response to positive stimulation were less often asymmetric than posed smiles (Ekman et al., 1981; Skinner and Mullen, 1991; Frank and Ekman, 1993; Frank et al., 1993; Hager and Ekman, 1997; Krumhuber and Manstead, 2009). However, Schmidt and colleagues have not found significant differences in the presence of asymmetry as a function of the spontaneous and deliberate production of smiles (Schmidt et al., 2006, 2009). It should be noted that in these latter studies spontaneous smiles were not induced by positive stimulations. Nevertheless, the results suggest that asymmetry is not a constant distinctiveness index between types of smiles. Furthermore, the presence of asymmetry in posed smiles was in some circumstances occasional (24% of deliberate smiles, Ekman et al., 1981), while in others more frequent (83% of deliberate smiles, Krumhuber and Manstead, 2009).

Several studies have demonstrated that the morphological characteristics associated with symmetry/asymmetry were used by adults in their appreciation of smiles. In fact, Chartrand and Gosselin (2005) found that asymmetric smiles were considered as less happy than symmetric smiles and less happy than smiles that did not include the Duchenne marker. Krumhuber and Manstead (2009) obtained similar results with less asymmetry being associated with higher ratings of genuineness and amusement. More importantly, in comparing the use of several physical characteristics in predicting how smiles are perceived, these latter authors found that along with the apex duration, asymmetry of the expression was a consistent predictor of how smiles were judged. One study by Gosselin et al. (2002b) did not observed that participants were sensitive to asymmetry as symmetric and asymmetric smiles were judged as equally happy. In sum, both with regards to asymmetry in the production of smiles and in its use as a cue in the judgment of smiles, more inconsistencies are observed in the literature than for the Duchenne marker. A possible explanation for these discrepancies is that less research has focused on this morphological cue.

One general observation from the judgment studies is that performance remains relatively modest. In effect, with regards to symmetry/asymmetry, while Chartrand and Gosselin (2005) observed that participants were sensitive to asymmetry, Gosselin et al. (2002b) did not. Furthermore, in studying the Duchenne marker, Frank et al. (1993) and Gosselin et al. (2002b) reported an average of 55% success rate, while Boraston et al. (2008) obtained performance level of around 70% for the same index. Manera et al. (2011) also highlighted the large individual variation in performance that has been observed in smile studies, performance typically ranging from around 30 to 100%. In sum, while in most studies participants seem sensitive to these cues as their performance is better than chance levels, the relatively low accuracy rates prompted research to explore reasons that prevent individual from being able to use the morphological cues distinguishing types of smiles more consistently.

## The perceptual-attentional limitations

One explanation that has been put forward to explain the difficulty encountered by the decoder in distinguishing types of smiles is attributed to perceptual-attentional limitations. In effect, when asked to explicitly report on changes that distinguish enjoyment and non-enjoyment smiles, only half of the participants indicated differences associated with the Duchenne marker and only one participant (out of 30) referred to the dimension of symmetry/asymmetry (Gosselin et al., 2002b). Furthermore, when presented with pairs of smiles and asked to say if smiles were similar or not, participant used the answer “different” significantly less often although the number of different and same trials were equal (Chartrand and Gosselin, 2005). Finally, participants mentioned changes in areas that did not vary as a function of the type of smiles (Gosselin et al., 2002b; Chartrand and Gosselin, 2005). While these finding lend support to the perceptual-attentional limitations hypothesis, Chartrand and Gosselin (2005) reported a mediation analysis revealing that the detection of the facial parameters did not significantly explain ability to judge the enjoyment of smiles. Authors concluded that other factors might play a more important role in distinguishing types of smiles (e.g., explicit knowledge).

While it may be possible that other factors are better suited to account for the difficulties in distinguishing smiles, further examinations are required before ruling out the importance of the perceptual-attentional factors. First, support for the perceptual-attentional limitation hypothesis in previous studies comes from indirect and *post-hoc* tasks. In effect, after the judgment task in Chartrand and Gosselin (2005), participants were required to detect similar and different pairs of smiles. While this strategy is innovative, it is limited because it does not allow a simultaneous examination of looking behavior during the judgment task. Second, evidence from previous studies also supposes that participants are conscious of their eye movements. For instance, Gosselin et al. (2002b), in asking participants to indicate where they observed the differences assume that participants were, in fact, using these same areas to make their judgment, which might not be the case.

A way to address these issues is to monitor eye movements simultaneously during the judgment task. Many researchers have maintained that eye movements, regardless of whether they occur reflectively or voluntarily, are natural indices of attentional-perceptual processing (see e.g., Posner, 1980; Rayner, 2009). In other words, basic eye movements such as moving from one target in space to another and then stopping were believed to result from shifts in attention by the observer to process the perceptual information available in the foveal region. A few studies have examined eye movements in relation to smile processing. Williams et al. (2001) conducted a study to examine patterns of eye fixations in order to better understand the visuo-cognitive strategies that underpin the perception of the Duchenne smile. Results revealed that participants made greater number and longer fixations to the Duchenne region (e.g., crow's feet) for happy expressions compared to sad and neutral expressions; possibly indicative of a natural tendency to focus on that specific marker when exposed to smiles. However, participants were asked to categorize stimuli as a function of emotion and were not asked to judge the nature of smiles. Consequently, results should be interpreted with caution because emotional categorization and appreciation of the smile might involve different mechanisms. Moreover, morphological characteristics were not manipulated in this study, making it difficult to reach conclusions about the processing of a specific feature.

Boraston et al. (2008) investigated the ability of adults with autism to distinguish enjoyment and non-enjoyment smiles while using eye-tracking. Because the Duchenne marker is characterized by appearance changes in the eye region, authors hypothesized that a reduced tendency to look at the eye region might lead to reduced ability to discriminate smiles as a function of this feature. Results revealed that adults with autism were impaired in the judgment task compared to the control group and, in addition, the former spent significantly less time in the eye region and made significantly fewer fixations to this region. The authors referred to these results as a support for the perceptual-attentional hypothesis with regards to the Duchenne marker. However, correlations between accuracy in the judgment task and the percentage of gaze time in the eye region were not significant, thus, shedding doubt on the role of perceptual-attentional mechanisms in the judgment of smiles.

In the same line, Manera et al. (2011) also investigated the relationship between perceptual-attentional factors in the recognition of enjoyment smiles based on the Duchenne marker. They used eye movement recording and tested the ability to discriminate appearance changes in the eye region (Duchenne marker or AU6 and Lid tightener or AU7) to examine to what extend perceptual-attentional factors explains individual differences in smile recognition. Eye movement data revealed that participants spent significantly more time in the eye region compared to the mouth region, especially when the Duchenne marker and the Lid tightener were activated, compared to smiles with neutral eyes; suggesting that individual are sensitive to the appearance changes created by muscular activation. However, neither correlation nor path analysis revealed that perceptual-attentional factors played a significant role in explaining individual differences in smile recognition. While these results seem to reject the perceptual-attentional limitations hypothesis, the analyses were not computed in a way to provide a clear comparison of the differences between enjoyment and non-enjoyment smiles. More precisely, accuracy was combined for all types of smiles. Consequently, differences might be observed between the processing of the enjoyment smile (with AU6) and both types of non-enjoyment smiles (with neutral eyes AU0 and with AU7). Furthermore, the results from eye movement measures showed that participants spent more time in the eyes when activation was present regardless of if this activation was congruent with enjoyment (AU6) or associated with non-enjoyment (AU7), thus, results with regards to the role of perceptual-attentional mechanisms as a function of enjoyment and non-enjoyment smiles remains obscure.

Recently, Calvo et al. (2013) investigate the question of congruency of the eyes with the smile. More precisely, they examined the visual attention patterns for smiles for which the eyes were congruent with the smile (the Duchenne smile) and smiles for which the eyes were incongruent. These incongruent smiles included the non-Duchenne smile (neutral eyes or AU0) and eyes conveying anger, fear, disgust, surprise, or sadness (blended smiles). The results revealed that participants judged the Duchenne smile as being happier than all blended smiles, including the non-Duchenne smile. With regards to eye movements, results revealed that participants spent more time in the mouth than the eyes. However, they spent more time in the eyes when they were incongruent (blended or neutral eyes) than when information was congruent with the smile (Duchenne smile). As Manera's et al. study did not allow the distinctions with regards to congruency, Calvo et al's results do not allow distinctions between activation and non-activation. In effect, within their study, the non-Duchenne smile for which the eyes were not activated was included with the blended smiles that did contain activation. In sum, in the latter two studies congruency and activation are confounded, thus with regards to the perceptual-attentional processing of smiles as a function of enjoyment and non-enjoyment requires further investigation.

The aim of the current study was to conduct a systematic examination of perceptual-attentional mechanisms used in distinguishing enjoyment and non-enjoyment smiles. More specifically, we explore the role of two indices reported in the literature on the production of smiles: the Duchenne marker and the symmetry. Furthermore, we directly explored eye movements simultaneously during the judgment task without relying on explicit recall while controlling for the confusion between congruency and activation.

Based on previous research, it can be predicted that participants will judge the Duchenne smile as happier than the non-Duchenne smile (Gosselin et al., 2002b; Chartrand and Gosselin, 2005). However, with regards to asymmetry, studies have been inconsistent, thus, the asymmetric smile might be judged as less happy than the Duchenne smile (Chartrand and Gosselin, 2005; Krumhuber and Manstead, 2009) or as equally happy (Gosselin et al., 2002b). With regards to the Duchenne marker, if individuals are sensitive to the activation of this index, this would be reflected in more time viewing the eye area where appearance changes are observed when smiles involve the activation of the Cheek raiser (Boraston et al., 2008; Manera et al., 2011). In other words, more time would be spent in the eye area for a Duchenne smile as well as an asymmetric smile than for a non-Duchenne smile since the former two include appearance changes associated with the Cheek raiser activation. However, based on Calvo's et al. (2013) results, if participants are sensitive to the incongruency of the smile with the information in the eyes, more time would be expected in the eye area for both types of non-enjoyment smiles (non-Duchenne and asymmetric). The sensitivity to asymmetry of smiles was examined using the number of saccades made from one side of the face to the other. If individuals show difficulty in perceptually distinguishing smiles that are asymmetrical compared to symmetrical, this would be reflected in similar patterns of comparison of the both sides of the face. In other words, difficulty would be reflected by an absence of difference for this index. In order to fully examine the perceptual-attentional hypothesis, relationships between the performance at the judgment task and an eye movements measures will be explored. If perceptual-attentional mechanisms are important in the performance, it will be reflected in a positive relationship between the measures.

## Method

### Participants

Six individuals (3 women, 3 men) were recruited as encoders to produce the facial expression stimuli used in the experiment. Thirty-two undergraduate students (28 women, 4 men; mean age 23 years old) participated in the experiment as decoders. All decoders reported normal or corrected-to-normal vision. All encoders and decoders were Caucasian. Participants were treated in compliance with ethical standards in effect at Laurentian University and only those who signed the informed consent took part in the study.

### Materials

**Figure 1:**
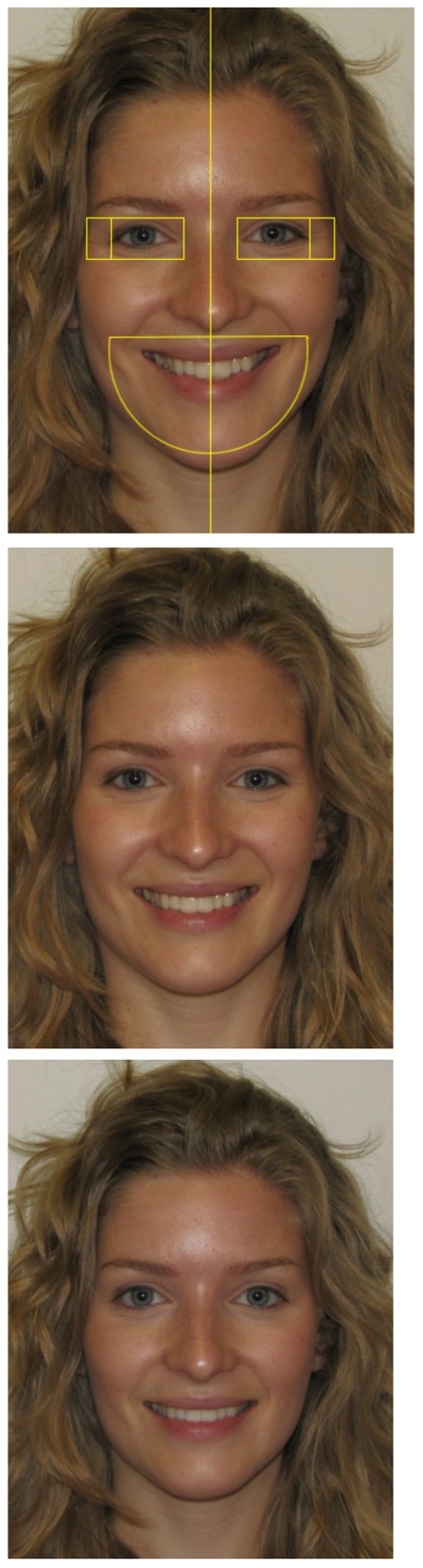
**An example of a symmetric Duchenne smile (Cheek raiser and Lip corner puller activated symmetrically) is presented in the upper panel, one with asymmetric activation is presented in the middle panel, and non-Duchenne smile without Cheek raiser activation is presented in the lower panel**. Examples of the zones are superimposed on the symmetric Duchenne smile.

The materials consisted of a series of pictures of smiling facial expressions developed according to criteria from the FACS (Ekman et al., 2002). The FACS defines 44 facial action units (AU) producing appearance changes in the activation of facial muscles. Two AUs were used in the production of the smiles: the Lip Corner Puller and the Cheek raiser or Duchenne marker. The FACS also includes norms for coding of the intensity of activation. Five levels of intensity are described from A (low intensity) to E (extreme intensity). The intensity is coded independently for each side of the face. The first smile included both the activation of the Lip Corner Puller and the Cheek raiser at intensity D. This smile will be referred to hereafter as the *symmetric Duchenne* smile. The second smile again included the activation of both AUs but the intensity varied from one side of the face to the other (C vs. D). For each encoder, in half of the pictures the right side of the face had a more intense activation and in the other half the left side of the face was more intensely activated. This smile will be referred to as the *asymmetric* smile. Finally, the third smile included only the activation of the Lip Corner Puller at intensity D on both sides of the face (see Figure 1 for examples of smiles). This smile will be referred to as the *non-Duchenne* smile. These stimuli were produced by the encoders in a single laboratory session conducted under the supervision of a FACS coder. It should be noted that the stimuli are produced artificially in laboratory to present specific manifestation of indices, while controlling others parameters^1^. In order to ensure that the pictures respected these criteria, they were assessed by two independent certified FACS judges. Prototypes with a perfect inter-rater agreement were used in the study. Ninety-six trials were presented to decoders: 48 enjoyment smiles (symmetric Duchenne smiles) and 48 non-enjoyment smiles (24 non-Duchenne and 24 asymmetric). The symmetric Duchenne smile was presented eight times for each of the six encoders for a total of 48 trials. The non-Duchenne smile was presented four times for each encoder for a total of 24 trials. Finally, the asymmetric smile was presented twice with the strongest intensity on the left and twice with it on the right for a total of 24 trials.

### Apparatus

Eye movements were recorded with the Eyelink II system. This apparatus is a highly accurate system (<0.5°) and has a high sampling rate (500 Hz). The apparatus has two cameras located under the participants' eyes and an infrared sensor located on the forehead. The forehead sensor allows head tracking for head movement compensation. One pupil was tracked in the current study and eye selection was determined by the most accurate calibration between the two pupils. A nine-point calibration was used and a maximum deviation of 1° in visual angle between both calibrations was deemed satisfactory. After calibration was established, participants were exposed to the stimuli on a 21″ ViewSonic monitor and at the same time, the experimenter's monitor displayed the participant's gaze position. The gaze position was displayed by a 1° in diameter gaze cursor, which allows examination of the system's accuracy.

### Procedure

Each participant was tested in one session lasting ~30 min. Participants were informed that 96 pictures of smiles would be presented one by one on the computer screen. They were informed that they would have to judge de sincerity of these smiles. They were instructed that, when they were confident of their answer, they should press the mouse button after which they had to respond verbally whether the person in the picture was “really happy” or “not really happy” (see Gosselin et al., 2002b; Del Giudice and Colle, 2007, for identical judgment task). After they provided their answer, the next picture was presented. Pictures were presented randomly for all participants.

### Data analyses

For all analyses an alpha level of 0.05 was used. The probability of answering “really happy” was computed for each type of smile (symmetric Duchenne, asymmetric and non-Duchenne) by dividing the number of times a participant answered “really happy” by the number of occurrences of each type of smile. An analysis was also conducted on expected responses. In other words, participants were expected to answer “really happy” for the symmetric Duchenne smile and “not really happy” for the other two types of smiles. The probability of producing the expected response was computed for each type of smile by dividing the number of expected responses by the number of occurrences of each type of smile. For the asymmetric smile, the prototypes were combined regardless of whether the more intense activation was on the left or the right side because supplementary analyses revealed no difference between these two types of smile on any of the dependent measures^2^.

Perception of discriminating indices was observed using eye movement measures. Eye movements were scored with the EyeLink Dataviewer. This program presents participants fixations superimposed on presented stimuli. For each type of smiles, the proportion of time spent on the eyes, the mouth, and crows' feet was computed by dividing the time spent in the specified zone by the total time spent on the stimulus. The size of the eye zone is ~2.48 by 1.24° in visual angles, the crows' feet zone 0.86 by 1.24 and the mouth zone 5.94 by 3.62 (see Figure 1 for examples). At least one fixation had to occur in the zone for an observation to be computed, without which an empty cell was recorded. It should be noted that presentation time was under the participants' control (range from 328 to 37,915 ms). Consequently, proportions were a more appropriate measure than total dwell time because it controlled for the important variations in viewing times. Nevertheless, analyses were also computed on dwell time. An analysis was computed on the total viewing time as a function of the type of smile. Total viewing time was computed by adding all fixation durations on the stimulus from the onset of its presentation on the screen to its disappearance. Finally, numbers of saccades from one side of the face to the other were computed. More specifically, each time the participant's eye crossed an invisible vertical boundary in the middle of the stimulus a saccade was counted regardless of whether the movement was from right to left or left to right.

## Results

### Answers

#### Answering “really happy”

**Figure 2:**
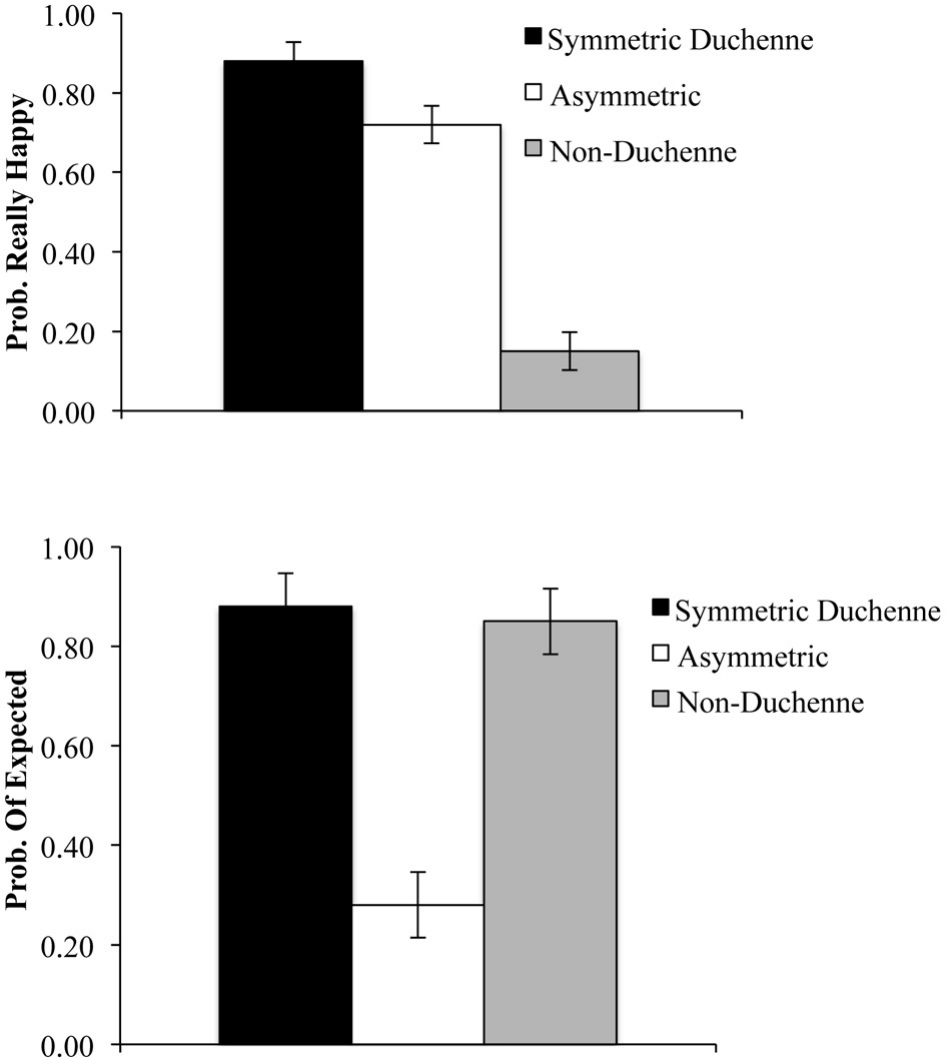
**Probability of answering “Really Happy” (upper panel) and probability of producing the expected response (lower panel) as a function of the type of smiles**. Error bars represent within-subject confidence intervals, at alpha level of 0.05, computed according to Loftus and Masson (1994). Differences are significant when error bars do not overlap by more than half their length.

A repeated-measures analysis of variance (ANOVA) revealed a significant effect of smile type, *F*_(2, 62)_ = 247.48, η^2^_*p*_ = 0.90, *p* = 0.001, (see upper panel of Figure 2). *Post-hoc* tests (Tukey) revealed that participants responded significantly more often “really happy” for the symmetric Duchenne smile than for asymmetric and non-Duchenne smiles and significantly more often for the asymmetric than for the non-Duchenne smiles.

#### Producing the expected response

Results revealed that participants produced the expected responses significantly more often for the symmetric Duchenne and non-Duchenne smiles than for the asymmetric smile (see lower panel Figure 2). These trends were confirmed by a repeated-measures ANOVA, *F*_(2, 62)_ = 106.54, η^2^_*p*_ = 0.78, *p* = 0.001. *Post-hoc* tests (Tukey) revealed that participants produced the expected response more often for the symmetric Duchenne and non-Duchenne smiles than for the asymmetric smile. The former two did not differ significantly.

### Eye movement measures

#### Total viewing time

**Figure 3:**
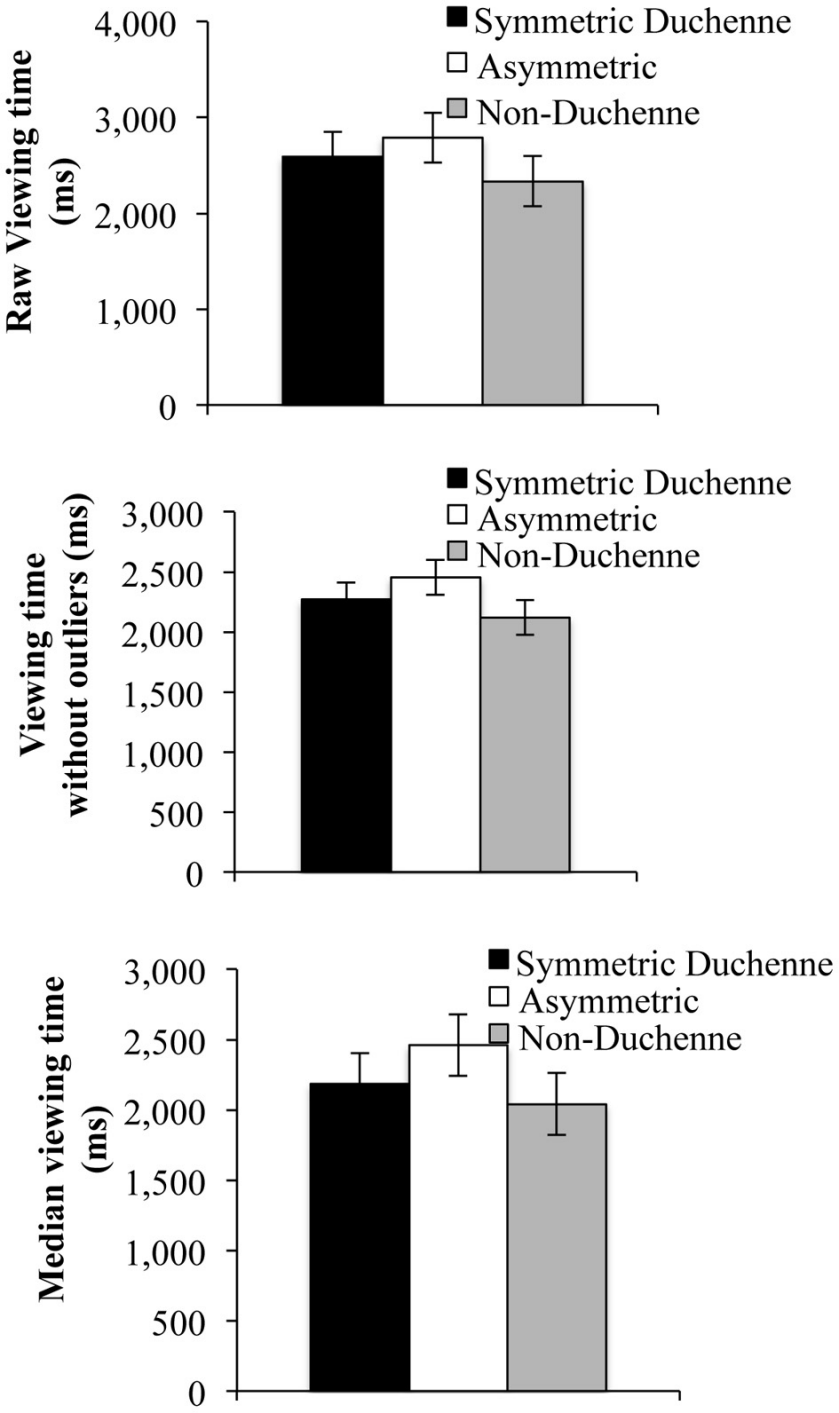
**Raw mean viewing time (upper panel), Mean viewing time without outliers (middle panel) and median viewing (lower panel)**. Error bars represent within-subject confidence intervals, at alpha level of 0.05, computed according to Loftus and Masson (1994). Differences are significant when error bars do not overlap by more than half their length.

Participants spent similar amounts of time looking at all types of smiles. The repeated-measures ANOVA was not significant, *F*_(2, 62)_ = 3.03, η^2^_*p*_ = 0.09, *p* = 0.06 (see upper panel Figure 3). However, when data were screened removing occurrences two standard deviations above or below the mean for each type of smile, results were significant, *F*_(2, 62)_ = 5.41, η^2^_*p*_ = 0.15, *p* = 0.01, (see middle panel of Figure 3) and again when the median was used instead of the mean, *F*_(2, 62)_ = 3.74, η^2^_*p*_ = 0.11, *p* = 0.03, (see lower panel of Figure 3). More precisely, *post-hoc* tests (Tukey) revealed that participants spent more time viewing the asymmetric smile than the symmetric Duchenne and non-Duchenne smiles. The latter two did not differ significantly.

#### Time spent in interest areas

**Figure 4:**
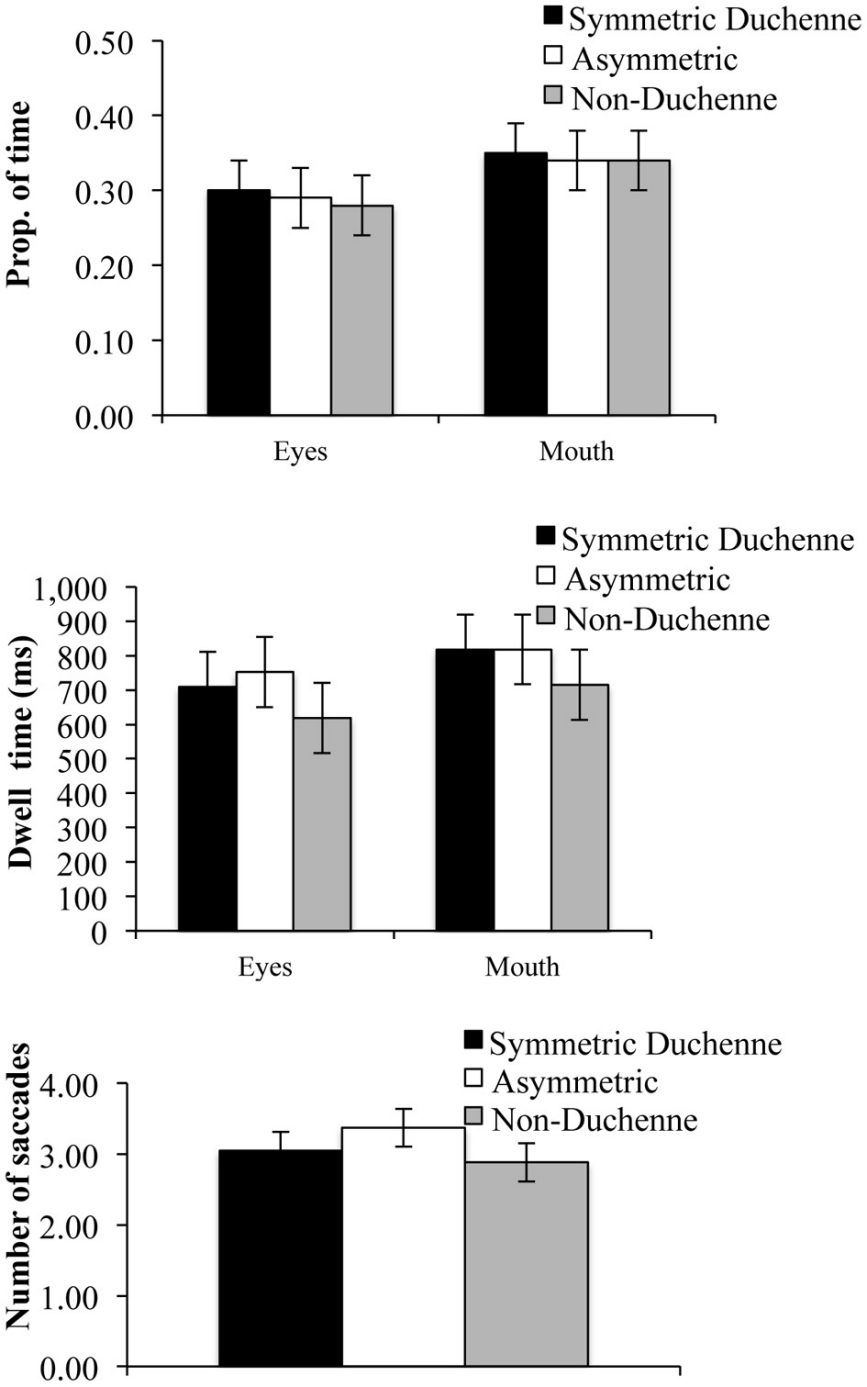
**Proportion of time spent in the eyes and mouth (upper panel), dwell time in the eyes and mouth (middle panel) and the number of saccades between each side of the face (lower panel)**. Error bars represent within-subject confidence intervals, at alpha level of 0.05, computed according to Loftus and Masson (1994). Differences are significant when error bars do not overlap by more than half their length.

Results revealed no obvious difference between the proportion of time spent in the eyes zone or the mouth zone as a function of the type of smile (see upper panel of Figure 4). The repeated-measures ANOVA with type of smile (symmetric Duchenne, asymmetric, and non-Duchenne) and zone (eyes and mouth) as within-subject factors revealed no main effect of type of smile, *F*_(2, 62)_ = 1.53, η^2^_*p*_ = 0.05, *p* = 0.23, no main effect of zone, *F*_(2, 62)_ = 0.99, η^2^_*p*_ = 0.03, *p* = 0.33, and the interaction was not significant, *F*_(2, 62)_ = 2.70, η^2^_*p*_ = 0.08, *p* = 0.08. A similar pattern of results was observed for dwell time (see middle panel of Figure 4): main effect of type of smile, *F*_(2, 62)_ = 1.68, η^2^_*p*_ = 0.05, *p* = 0.19, main effect of zone, *F*_(2, 62)_ = 0.42, η^2^_*p*_ = 0.01, *p* = 0.52, and interaction, *F*_(2, 62)_ = 1.88, η^2^_*p*_ = 0.06, *p* = 0.16.

An analysis was also computed for the proportion of time spent in the crows' feet zone as a function of type of smile. It should be noted that the analysis is conducted on only 9 participants because 23 participants did not fixate on the crows' feet for at least one type of smile. No significant difference was observed, *F*_(2, 16)_ = 0.13, η^2^_*p*_ = 0.02, *p* = 0.88 (symmetric Duchenne *M* = 0.11, *SD* = 0.04; non-Duchenne *M* = 0.10, *SD* = 0.05; asymmetric *M* = 0.12, *SD* = 0.10). A similar pattern of results was observed for dwell time, *F*_(2, 16)_ = 0.09, η^2^_*p*_ = 0.01, *p* = 0.92 (symmetric Duchenne *M* = 315 ms, *SD* = 73; non-Duchenne *M* = 328 ms, *SD* = 140; asymmetric *M* = 336, *SD* = 109).

#### Saccades between sides of the face

Results for the number of saccades between the two sides of the face as a function of type of smile (symmetric Duchenne, non-Duchenne, and asymmetric) revealed that participants made more saccades from side to side for the asymmetric than for the other two types of smiles (see lower panel of Figure 4). These trends were confirmed by a repeated-measures ANOVA, *F*_(2, 62)_ = 3.49, η^2^_*p*_ = 0.10, *p* = 0.03. *Post-hoc* tests (Tukey) revealed that participants made more saccades from one side of the face to the other for the asymmetric smile than for the symmetric Duchenne and non-Duchenne smiles. The latter two did not differ significantly.

#### Correlations

A series of correlations were computed between the proportion of expected responses and the measures of eye movements (proportion of time in the eyes, crows' feet, and mouth; the dwell time in the eyes, crows' feet, and mouth; number of saccades; mean viewing time without outliers and median viewing time) considering all available data for all participants. None of the correlations were significant. For sake of brevity, the highest correlation was with the proportion of time spent in the crows' feet, *r* = −0.40, *p* = 0.07.

Partial correlations were also computed between these same variables while controlling for the type of smile. This time, the correlation between proportion of expected response and proportion of time spent in the crows' feet was significant, *r* = −0.30, *p* = 0.04. More specifically, the more time participants spent in the crows' feet, the lower the accuracy. This was not the case when dwell time was considered instead of proportion of time, *r* = −0.06, *p* = 0.70. In fact, none of the other partial correlations were significant. Nevertheless, because of these previous results, patterns were examined separately for each type of smile. These correlations revealed that for the asymmetric smile, the higher the proportion of time in the crows' feet, the lower the accuracy, *r* = −0.67, *p* = 0.003, but no relationship was observed for the symmetric Duchenne, *r* = 27, *p* = 0.26 or the non-Duchenne smiles, *r* = −0.12, *p* = 0.69.

## Discussion

The current study aimed at better understanding the role of perceptual-attentional mechanisms in the processing of morphological cues associated with enjoyment and non-enjoyment smiles. This was accomplished by exploring eye movements in a smile recognition task. We extended on previous literature by not only examining the importance of the Duchenne marker but also the symmetry of activation of morphological cues. Furthermore, we aimed at clarifying discrepancies the literature both with regards to the judgment task but also to eye movement recordings in smile processing. In effect, it remained unclear how individuals respond to asymmetry (Gosselin et al., 2002b; Chartrand and Gosselin, 2005). Furthermore, with regards to eye movement research, it also remained ambiguous if participants were sensitive to the activation of facial muscles or to the incongruency between activation of the mouth and the eyes (Manera et al., 2011; Calvo et al., 2013). Finally, we explored the link between the performance at the judgment task and viewing behavior in order to provide insight in the debate on the role of perceptual-attentional mechanisms in the appreciation of smiles.

### The duchenne marker

In the judgment task, participants judged the symmetric Duchenne smile as happier than the non-Duchenne smile. The current results also showed that participants are effective in judging the absence of the Duchenne marker as a sign of non-enjoyment. These results are in line with previous studies showing that adults process to this index and that they, in fact, interpret the absence of activation of this muscle as a sign of non-enjoyment (Frank et al., 1993; Manera et al., 2011; Gosselin et al., 2002b; Chartrand and Gosselin, 2005; Leppänen and Hietanen, 2007; Miles and Johnston, 2007; Ambadar et al., 2009; Krumhuber and Manstead, 2009; Thibault et al., 2009, 2012; Calvo et al., 2013).

The novel finding in this study, with regards to the judgment responses, was the extent of the sensitivity to this index. More precisely, results showed no evidence of difficulty associated with the expected interpretation of either the presence or absence of the Duchenne marker. In effect, the accuracy in the judgment for the non-Duchenne smile was 85% and for the Duchenne smile, 88%. Contrary to previous studies, no indication of difficulty was observed in judgment of the smiles that vary as a function of the activation of the Cheek raiser (e.g., Frank et al., 1993; Gosselin et al., 2002b; Chartrand and Gosselin, 2005; Miles and Johnston, 2007).

With regards to viewing behavior, if individuals perceptually process to the activation of the Duchenne marker, this would be reflected in more time viewing the eye area where appearance changes are observed when smiles involve this activation (Boraston et al., 2008). No differences were found in the time spent in the eye region between the types of smiles. More precisely, participants did not spend more or less time in the eye area whether the Cheek raiser was activated or not. These results do not support the perceptual-attentional limitation hypothesis (e.g., Gosselin et al., 2002b; Chartrand and Gosselin, 2005). In effect, since participants process the eye area equally for all types of smiles, the difficulty observed in previous research could not be associated, here, with lack of time spent in the informative region (Williams et al., 2001; Boraston et al., 2008). This is further supported by the absence of relationship between the performance at the judgment task and eye movement measures.

In sum, the high levels of performance in the judgment task and absence of differences in eye movement patterns between the Duchenne and non-Duchenne smiles do not support the idea that there are perceptual difficulties or even difficulties in the interpretation of the Cheek raiser activation. In effect, not only do the current results question the perceptual-attentional limitation hypothesis with regards to the processing of the Duchenne marker, but they do not suggest difficulty at all (Manera et al., 2011). Both the judgment performance and looking behavior support the sensitivity to this index. A possible theoretical explanation of the results could be that the processing of the eye area would reflect an attentional preference that is acquired rapidly, which allows individuals to effectively recognize a relevant signal of enjoyment as an indicator of the emotional state of happiness (Owren and Bachorowski, 2001; Williams et al., 2001; Chartrand and Gosselin, 2005).

### The asymmetry

While results for the Cheek raiser activation do not suggest difficulty with the use of this index, the same is not as evident for asymmetry. Participants judged this latter smile as less happy than the symmetric Duchenne smile. However, in terms of performance, they still judged asymmetric smiles as really happy most of the time (72%). Thus, the results suggest a difficulty associated with the judgment of asymmetry. These results are in line with previous studies (Gosselin et al., 2002b; Chartrand and Gosselin, 2005).

Based only on the judgment task, it is impossible to discriminate if the difficulty is due to perceptual-attentional limitations or interpretation of the index. Thus, we also examined looking behavior. In effect, eye movements showed a differential processing of the asymmetric smile compared to the two other types of smiles. More specifically, participants made more saccades from one side of the face to the other when the smile was asymmetric. This suggests that they perceive differences between the types of smiles.

Consequently, the results for eye movement patterns do not support the perceptual-attentional limitation hypothesis (Gosselin et al., 2002b; Chartrand and Gosselin, 2005; Manera et al., 2011). Since participants do process the smiles differently, we can infer that they perceive differences. Nevertheless, participants spent more time viewing this type of smile than the others suggesting some degree of difficulty in its processing.

In sum, the differences in eye movement behaviors and the low performance at the judgment task reflect difficulty associated with the interpretation of the asymmetry rather than to perceptual-attentional limitations. In line with Chartrand and Gosselin's (2005) explanations, these results suggest that with regards to this specific characteristic of non-enjoyment there are other factors involved rather than simply viewing this specific sign. For instance, while participants process symmetric and asymmetric smiles differently, it could be hypothesized that participants might not interpret this change as a sign of non-enjoyment. Furthermore, for this type of smile, correlations suggest that participants might be biased by the activation of the Duchenne marker. In effect, the more time they spent fixating the crows' feet area where appearance changes associated with this cue are shown, the higher the probability of judging the smile as happy. Further research is necessary to explore the explicit knowledge associated with the asymmetry of smiles.

A possible explanation for these results could be that social experiences that allow the acquisition of knowledge related to the interpretation of asymmetry as a sign of non-enjoyment might not be frequent (Chartrand and Gosselin, 2005). In effect, Ekman et al. (1981) reported that asymmetry was occasional. Furthermore, in order for the individual to develop the knowledge relative the asymmetry as a sign on non-enjoyment in smiles, he or she should be exposed to situations where it is possible to observe the contingency between the asymmetry of the expression and other indices revealing that the person does not feel truly happy. As suggested by Ekman et al. (1981), it is possible that this type of situation is rare.

Another explanation that may prevent the acquisition of this knowledge resides in the presence of some degree of asymmetry in spontaneous expression of emotions. In a comprehensive review, Borod et al. (1997) observed that the left side of the face is more involved than the right side in the expression of emotions regardless of valence. Nevertheless, in the expression of happiness, asymmetry is still more often associated with simulated than spontaneous smiles. It might be the general knowledge of emotional facial expressions that would conflict with the specific knowledge for expressions of happiness. In other words, asymmetry might be viewed as a sign of spontaneity because it is one in other circumstances.

Finally, the current study also allowed the examination of the extent to which the activation of morphological cues or the incongruency between the smile and the information in the eye area influence viewing behavior. In effect in previous research these factors were confounded (Manera et al., 2011; Calvo et al., 2013). When these factors are controlled, neither the activation nor incongruency accounts for viewing behavior since no differences were found in the time spent in the eyes or mouth as a function of these factors. On the one hand, it should be noted that the morphological cues used in the current study were different from those in previous research. Consequently, results seem to vary as a function of the nature of the activation. On the other hand, in comparison to the previous studies, the morphological cues used in the current study correspond to indices observed in the production of smiles, while those in the previous studies (Manera et al., 2011; Calvo et al., 2013) include signs that are not reported as non-enjoyment (e.g., the Lid tightener or blended smiles). Nevertheless, further research is necessary to explore this question.

### Limitations

One aspect of this study might be viewed as a limitation that could prevent the generalization of the results. The sample is constituted of an unbalanced gender representation. This is due to the sampling pool recruited in undergraduate psychology classes, which are constituted of a majority of females. However, previous research in this field has failed to find any indication of gender differences in the judgment of enjoyment and non-enjoyment smiles (e.g., Frank et al., 1993; Thibault et al., 2009) and more precisely, when exploring the specific indices used in the current study (Gosselin et al., 2002b). Furthermore, even in studies with children or adolescents, there were still no gender differences (Thibault et al., 2009; Gosselin et al., 2010b). Thus, there was no reason to suspect gender differences with regards to the judgment task. Nevertheless, an eye movement study in the recognition of facial expressions of basic emotions has shown that while there is no difference in the time spent viewing the eyes, males spent more time viewing the mouth and nose than females (Vassalo et al., 2009). Future research should consider using equal groups in terms of gender.

Future research in the judgment of smiles should also take into consideration another variable that could influence the interpretation of the results: handedness. This variable would be of interest particularly with regards to judgment of asymmetric expressions. Since handedness was not recorded in the current study, it might be viewed as a limitation. In effect, in the context of studies pertaining to hemispheric specialization, results have suggested that right-handed individuals have a bias toward the left side of the face, which is less pronounced for left-handed individuals (see e.g., Luh et al., 1991). However, it should be noted that handedness differences are not always observed with regards to emotional facial expression processing (Van Strien and Van Beek, 2000). Nevertheless, while it might have been interesting for the interpretation of the current results, it does not invalidate the current observations.

## Conclusion

The main finding of the current study is that, while the activation of the Duchenne marker is a more effective factor in judgment of enjoyment and non-enjoyment smiles than asymmetry, the differences between the judgment responses are not associated with perceptual-attentional limitations. Consequently, the perceptual-attentional limitation hypothesis is not a satisfactory explanation for difficulties in the judgment of smiles. It seems that humans possess the ability to perceive subtle details of facial expressions. Future research should explore the role of explicit knowledge and its development with regards to the signification of these types of indices. Furthermore, attention should be given to looking behavior as well as interpretation of other indices pertaining to the nature of the smiles such as micro-expressions associated to hidden negative emotions (see e.g., Ekman, 2003, 2009; Porter et al., 2012).

### Conflict of interest statement

The authors declare that the research was conducted in the absence of any commercial or financial relationships that could be construed as a potential conflict of interest.

## Footnotes

^1^Following Chartrand and Gosselin's (2005) and Gosselin et al.'s (2002b) reasoning, while stimuli are artificial and do not represent expressions underlying real emotions, we stipulate that if participants are able to perceive and interpret the indices in real life, this would be manifested in the current task. For instance, if they perceive the indices of enjoyment in our pictures, we expect participants to judge them as happier than when they contain signs of non-enjoyment. Based on this assumption, results are analyses as a function of the tendency to response “really happy” as well as a function of expected response.

^2^Supplementary analyses were conducted on the asymmetric smiles as a function of the side (left or right) for which the activation was more intense. In effect, studies have indicated a bias or a tendency to focus on the left side of the face in judgment of facial stimuli (e.g., Heller and Levy, 1981; Levy et al., 1983; Borod et al., 1990). Dominance of the left half of faces is explained by the projection primarily to the right hemisphere, which is considered as specialized for expression and appreciation of emotion (Borod et al., 1983; Silberman and Weingartner, 1986). From these studies, one might suspect a greater percentage of really happy responses for the asymmetric faces where the left half face has a greater intensity of emotional expression (D) rather than for the asymmetric faces in which the left side of the face has a lower intensity of emotional expression (C). The same applies for the analysis of the left-right saccades. We have computed analyses on proportion of answering “really happy,” proportion of expected responses, the number of saccades, the proportion of time and dwell time spent on the crows' feet, eye, and mouth areas. None of these analyses were significant, all *F*s < 1.84, η^2^_*p*_< 0.24, *p* > 0.22.
